# Complete Genome Sequence of *Herbaspirillum hiltneri* N3 (DSM 17495), Isolated from Surface-Sterilized Wheat Roots

**DOI:** 10.1128/genomeA.01288-15

**Published:** 2015-10-29

**Authors:** Dieval Guizelini, Paula M. Saizaki, Nilson A. R. Coimbra, Vinicius A. Weiss, Helisson Faoro, Michelle Z. T. Sfeir, Valter A. Baura, Rose A. Monteiro, Leda S. Chubatsu, Emanuel M. Souza, Leonardo M. Cruz, Fabio O. Pedrosa, Roberto T. Raittz, Jeroniza N. Marchaukoski, Maria B. R. Steffens

**Affiliations:** a Department of Biochemistry and Molecular Biology, Federal University of Paraná, Curitiba, PR, Brazil; b Laboratory of Bioinformatics, Professional and Technological Education Sector, Federal University of Paraná, Curitiba, PR, Brazil

## Abstract

We report the complete genome sequence of *Herbaspirillum hiltneri* N3 (DSM 17495), a member of the genus *Herbaspirillum* of the *Betaproteobacteria*. The genome is contained in a single chromosome, and analysis revealed that N3 lacks the whole nitrogen fixation (*nif*) gene cluster, confirming its inability to fix nitrogen.

## GENOME ANNOUNCEMENT

*Herbaspirillum hiltneri* N3 (DSM 17495) is a betaproteobacterium isolated from surface-sterilized wheat roots, first described by Rothballer et al. (1).

Although the genus *Herbaspirillum* contains diazotrophic species such as *H. seropedicae, H. rubrisubalbicans*, and *H. frisingensis*, with the potential for endophytic and systemic colonization of a variety of plants, *H. hiltineri* strain N3 lacks all genes of the *nif* cluster, confirming that this organism is unable to fix nitrogen, as previously described (Rothballer et al. 2006 [1]). However, the genes involved in the overall regulation of nitrogen metabolism (*glnA*, *glnB*, *glnE*, *glnK*, *ntrB*, *ntrC*, *ntrY*, and *ntrX*) are present.

The genome was sequenced using the SOLiD sequencing platform. Short reads in color space formatted from whole-genome shotgun sequencing (WGS) in fragments (8.4 million) and mate paired (107 million) were partially assembled using the *de novo* pipeline accessory tools 2.0. The best assemblies from each data set were integrated within a hybrid assembly by the assembler Phrap, resulting in 5,970 contigs and 283 scaffolds. To finish the genome sequence, a Nextera paired-end library 250 bp in length was sequenced on an Illumina MiSeq, producing 5,088,454 sequences. All reads were assembled with Velvet 1.2.07 (2), CLC Genomics Workbench 6.5.1, ALLPATHS-LG (3, 4), and Edena V3 (5). The resulting contigs were aligned to the reference genomes of *H. seropedicae* strain SmR1 (6) and *H. lusitanum* P6-12 (7) and, finally, the gaps were closed with FGAP (8).

The complete genome sequence of *Herbaspirillum hiltneri* N3 is 4,965,474 bp long with a G+C content of 61.84%. This genome is 548,413 bp smaller than that of *H. seropedicae* (6). Genome annotation using RAST (9) and our in-house SILA platform predicted 4,581 coding sequences (CDS), tRNAScan predicted 49 tRNAs, and NCBI Blast identified 4 16S-23S-5S rRNA operons.

As in *H. seropedicae* and *H. huttiense* subsp. *putei* IAM, the *H. hiltneri* genome contains the genes for nitrate transport and assimilation, namely, *nasA*, *nirD*, *nark*, and *nasFED*, while lacking all genes involved in the nitrate dissimilatory pathway *narG*, *narH*, *narI*, *narU*, and *narXL*. The gene coding 1-aminocyclopropane-1-carboxylate (ACC) deaminase, an enzyme purportedly involved in relieving ethylene-induced plant stresses, is also present, as in the previously described *Herbaspirillum* spp. genomes. The genome contains genes of the protein secretion systems type I, II, III, V, and VI. Type III has been suggested to be involved in plant–bacterium interactions.

The *H. hiltneri* genome contains all genes of the pentose phosphate pathway and the Entner-Doudoroff and Embden-Meyerhoff-Parnas (EMP) glycolytic/gluconeogenic pathways (except 6-phosphofructokinase [PFK], EC 2.7.1.11). As in *H. seropedicae*, *H. hiltneri* probably enrolls the Entner-Doudoroff and the pentose phosphate pathways to bypass the lack of PFK during glycolysis. The presence of isocitrate lyase supports gluconeogenesis from 2-carbon substrates such as acetyl coenzyme A (acetyl-CoA).

### Nucleotide sequence accession number. 

This whole-genome shotgun project has been deposited at DDBJ/EMBL/GenBank under the accession number CP011409. The version described in this paper is the first version.
